# Dissociable roles of medial and lateral PFC in rule learning

**DOI:** 10.1002/brb3.551

**Published:** 2016-08-19

**Authors:** Bihua Cao, Wei Li, Fuhong Li, Hong Li

**Affiliations:** ^1^ School of PsychologyJiangXi Normal UniversityNanchangChina; ^2^ School of Psychology and SociologyShengzhen UniversityShenzhenChina

**Keywords:** medial prefrontal cortex, rule discovery, rule learning

## Abstract

**Introduction:**

Although the neural basis of rule learning is of great interest to cognitive neuroscientists, the pattern of transient brain activation during rule discovery remains to be investigated.

**Method:**

In this study, we measured event‐related functional magnetic resonance imaging (fMRI) during distinct phases of rule learning. Twenty‐one healthy human volunteers were presented with a series of cards, each containing a clock‐like display of 12 circles numbered sequentially. Participants were instructed that a fictitious animal would move from one circle to another either in a regular pattern (according to a rule hidden in consecutive trials) or randomly. Participants were then asked to judge whether a given step followed a rule.

**Results:**

While the rule‐search phase evoked more activation in the posterior lateral prefrontal cortex (LPFC), the rule‐following phase caused stronger activation in the anterior medial prefrontal cortex (MPFC). Importantly, the intermediate phase, the rule‐discovery phase evoked more activations in MPFC and dorsal anterior cingulate cortex (dACC) than rule search, and more activations in LPFC than rule following.

**Conclusion:**

Therefore, we can conclude that the medial and lateral PFC have dissociable contributions in rule learning.

## 1 Introduction

Human behavior is often governed by rules that associate stimuli with responses (Badre, Kayser, & D'Esposito, 2010; Sakai, 2008; Waskom, Kumaran, Gordon, Rissman, & Wagner, 2014; Zhang, Kriegeskorte, Carlin, & Rowe, 2013). Many studies have shown that, during rule learning, the network of frontoparietal–temporal regions is selectively activated (Badre et al., 2010; Bunge, 2004; Bunge & Wallis, 2008; Hampshire, Thompson, Duncan, & Owen, 2011; Monchi, Petrides, Petre, Worsley, & Dagher, 2001; Reverberi, Görgen, & Haynes, 2012; Strange, Henson, Friston, & Dolan, 2001).

More recent studies have attempted to reveal the neural substrates underlying the subprocesses of rule learning (Crescentini et al., 2011; Seger & Cincotta, 2006; Tachibana et al., 2009). For example, Tachibana et al. (2009) found that the posterior medial frontal cortex and caudate nucleus were activated in response to a sequence rule or a probability rule. Crescentini et al. (2011) defined five phases of rule learning including search 1, search 2, discovery, following 1, and following 2. In the behavioral data, they reported the reaction time for each of the five phases. In the fMRI data, they emphasized on the difference between rule acquisition and rule following, and revealed that the mid‐dorsolateral prefrontal cortex (mid‐DLPFC) was more active during rule acquisition (i.e., rule search and discovery) than during rule following. In the ROI data, the rule‐discovery phase was compared to other phases, but the difference between rule discovery and rule search was not found at any cortex. In the study by Tachibana et al. (2009), brain activation found to be associated with rule discovery could not be isolated from the process of hypothesis testing.

In a rule identification task, Li, Cao, Gao, Kuang, and Li (2012) recorded the scalp potentials of participants at distinct stages of rule learning. Their results revealed that, compared with rule search, rule discovery elicited a larger P3 component. Based on the findings of Li et al. (2012), we hypothesized that there is a neuropsychological dissociation between rule discovery and rule search even though these two phases has been regarded as the same cognitive process (i.e., rule acquisition). To test this hypothesis, we adopted a modified rule attainment task based on previous studies (Crescentini et al., 2011; Li et al., 2012). In this task, participants were presented with clock‐like cards, each containing 12 circles with only one of them colored blue. Participants were informed that the blue circle would move from one position to another and that the movement may be congruent with a hidden rule. Participants were then asked to judge if the movement of the blue circle followed a rule or not.

Rule or regularity is often reflected by invariance of the relationship among sequentially presented objects (Cai, Li, Wang, & Li, 2014; Li et al., 2012). During rule‐search phase of rule learning, participants kept searching for a rule, but they did not detect the rule (or regularity). After a series of trials of rule searching, the rule or regularity among stimuli sequence was detected by participants, and this transient phase is referred as rule discovery (Crescentini et al., 2011; Li et al., 2012). Previous studies have demonstrated that PFC neurons encode abstract rules (Bongard & Nieder, 2010; Kamigaki, Fukushima, Tamura, & Miyashita, 2012; Vallentin, Bongard, & Nieder, 2012; Wallis, Anderson, & Miller, 2001). Specifically, medial prefrontal cortex (MPFC) has been suggested to be responsible for adaptive behavior, performance monitoring, number series completion, sequence learning, and mental set shifting (Destrebecqz et al., 2003; Euston, Gruber, & McNaughton, 2012; Qiu et al., 2010; Shen, Luo, Liu, & Yuan, 2013; Yang, Liang, Lu, Li, & Zhong, 2009; Zarr & Brown, 2016). Accordingly, we predicted that PFC, particularly the MPFC, would be more active in the rule‐discovery phase than that in the rule‐search phase.

On the other hand, during the rule‐search phase, participants should keep searching for invariance or regularity among stimuli or the predictive links between stimuli and response in order to generate hypothesis about the hidden rule. Previous studies have suggested that the lateral PFC is associated with the process of hypothesis generation (Goel & Vartanian, 2005; Goel et al., 2007; Boettiger & D'Esposito, 2005; ;Seger et al., 2000; Seger & Cincotta, 2006; Crescentini et al., 2011; Reverberi, D'Agostini, Skrap, & Shallice, 2005; Xiao, Li, Long, Lei, & Li, 2014). Therefore, we predicted that the lateral PFC would be more active during the rule‐search phase compared to discovery phases.

## 2 Methods

### 2.1 Participants

In total, 21 right‐handed healthy volunteers (10 males, 11 females, mean age = 21.4 years, *SD* = 1.53) participated in this study. All participants had normal or corrected‐to‐normal vision. No history of neurological or psychiatric diseases was reported by the participants. Functional magnetic resonance imaging (fMRI) data of one subject was excluded owing to excessive head movement. Each participant signed a consent form prior to the study. The study was conducted with full approval from the local Review Board for Human Participants Research of the Southwest University, China.

### 2.2 Stimuli and design

**Figure 1:**
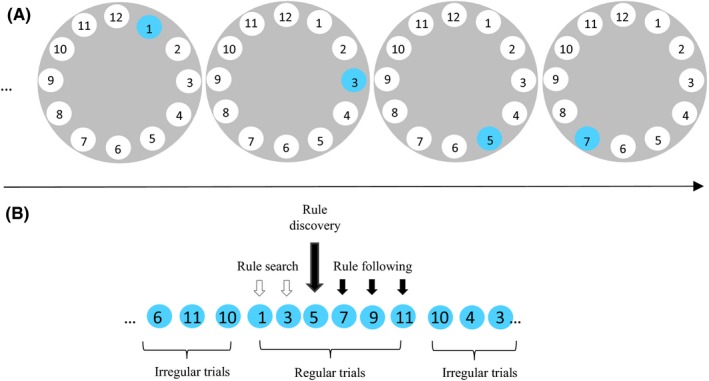
A trial of the task and the experimental design. Each rule consisted of a variable number of colored cards in which one circle was colored blue. Participants were required to press one of the two keys to indicate whether the blue circle in the current card was congruent with a hidden rule that pertains to the relationship between successive cards. (A) materials provided in one trial. (B) different types of trials and different kinds of regular trials that tapped into three phases of rule learning

A modified rule attainment task (Burgess & Shallice, 1996; Crescentini et al., 2011; Li et al., 2012) was used in this study. Participants were presented with a series of cards, each containing a clock‐like display of 12 circles numbered sequentially (Fig. 1). Among the 12 circles, one was colored blue, while the others were white. Participants were instructed that a fictitious animal would jump from one circle to another and that the circle under which the animal stood would turn blue. Participants were informed that the animal would jump according to a hidden rule during a series of consecutive trials, and jump randomly during other trials. That is, the location of the blue circle changed regularly across a series of trials (e.g., 1‐3‐5‐7‐9) and changed irregularly across other trials (e.g., 1‐3‐6‐2‐3‐11). Participants were asked to identify the hidden rule and judge whether the current location (blue circle) was congruent with a rule or not. They were required to respond by pressing one of two keys. For example, during the first half of the session, participants were asked to press keys F and J for rule‐congruent trials and rule‐incongruent trials, respectively, and press the opposite keys during the second half of the session.

Each rule was in operation from six to 11 regular trials (e.g., “+2” rule in Fig. 1), after which it changed without warning. A total of 20 different rules were adopted from the study of Li et al. (2012). The rules ranged from very easy, such as the “+1” rule, to more complex, such as “+1–2.” Each rule was applied twice in different examples (e.g., the number serials “3‐5‐7‐9” and “2‐4‐6‐8” are two examples of the “+2” rule) in different runs.

A total of 40 examples with 20 rules were organized in four different runs. The order of runs was counterbalanced across subjects. The difficulty of the rules and the number of cards within a run were balanced across runs. On average, each run lasted 12 min. Each run started with a total of one to five irregular trials (i.e., no rule was hidden in these trials), followed by 5–11 regular trials, then 1–5 irregular trials, and so on. In each run, approximately 65% of the trials were regular trials, while 35% were irregular trials. Since the number of regular and irregular trials always varied, participants could not guess the task.

The definition of rule phases was the same as that used in previous studies (Crescentini et al., 2011; Li et al., 2012). As was shown in Fig. 1, three types of regular trials were defined and each type of trial was meant to engage a different phase of rule learning: (1) rule search, (2) rule discovery, and (3) rule following. The regular trials that preceded the rule‐discovery trial and were correctly judged as rule‐incongruent trials were defined as rule‐search trials (Fig. 1). For instance, if participants were presented with the following stimuli, …4‐***1**‐3‐5‐7‐9‐11*….the number sequence with italic numbers is defined as regular trials with the hidden rule (+2). However, when participants were encountered with the first italic number (i.e., number 1), they can figure out the relationship between number 1 and the preceding number (e.g., 4), but they could not figure out the predetermined rule. When the second italic number (e.g., 3) was displayed, participants can also figure out the distance between current location and the preceding location, but the hidden rule is unlikely to be found before the presentation of the third italic number. Therefore, we defined the regular trials that preceded the rule‐discovery trial and were correctly judged as rule‐incongruent trials as rule‐search trials.

During some irregular trials, participants might keep searching for new rules, but we did not define all irregular trials as rule‐search trial because the process of rule violation is involved at some irregular trials (Li et al., 2012). For example, if participants were presented with the following sequence: 1‐3‐5‐7‐9‐11‐***12‐9‐10***‐, the solid and italic numbers in this sequence were irregular trials, but the number 12 is the violation of the rule +2, which is hidden in the “1‐3‐5‐7‐9‐11” serials. In order to eliminate the confounding of violation process with rule‐search process, we did not define all irregular trials as rule‐search trial.

The rule‐discovery phase was the first of the three successive trials in which participants correctly indicated that the positions of the blue circle followed a rule. The rule‐following phase included regular trials after the rule‐discovery trial that were judged correctly as rule‐congruent trials. It should be noted that there was no way for us to confirm that the subjects knew the rule correctly during the rule‐discovery and rule‐following phases, instead we could infer that they might discover the hidden rule if they correctly made three successive trials because the chance of guessing is lower than 12.5% (i.e., 1/2 × 1/2 × 1/2).

### 2.3 Procedure

Each colored card was presented for a maximum of 4 s. Participants were instructed to use the time to think about whether the position of the blue circle was congruent with a rule (i.e., whether the current trials is a rule‐congruent trial). They were asked to press one of the two keys to respond. For example, during half session, participants were asked to press keys F and J for rule‐congruent trials and rule‐incongruent trials, respectively, and press the opposite keys during the second half of the session. After pressing the key, the next colored card was presented after an intertrial interval of 2–6 s. Before the fMRI scan, all participants practiced the tasks for approximately 2 min.

### 2.4 fMRI image acquisition

Images were acquired using a 3‐Tesla Siemens fMRI instrument (Siemens, Erlangen, Germany). Blood oxygenation level‐dependent contrast was obtained using echo planar T2*‐weighted imaging (EPI). Acquisition of 32 transverse slices provided coverage of the whole cerebral cortex. Repetition time was 2 s, echo time was 29 ms, in‐plane resolution was 3.4375 × 3.4375 mm, and slice thickness and gap were 3 mm and 0.99 mm, respectively.

Stimuli were presented using a magnet‐compatible projector that back‐projected visual images onto a screen mounted above the participant's head. The experimental task was programmed using E‐Prime software. Responses were obtained using a magnet‐compatible response system.

### 2.5 Image processing

Functional images were preprocessed using SPM8 (Welcome Department of Cognitive Neurology, London, UK; RRID: SCR_007037) (Friston et al., 1995). Slice timing was used to correct the slice order. The data were realigned for estimating and modifying the six parameters of head movement. The first five images were discarded for achieving magnet‐steady images. The images were then normalized to Montreal Neurological Institute (MNI) space in 3 × 3 × 3 mm^3^ voxel size. The resulting set of transformations was applied to the participant's functional image volumes to form volume time course representations for use in subsequent statistical analyses.

All the events were modeled in the design matrix; however, the event of interest was neural activity evoked by regular trials, which were tapped into the three phases of rule learning (search, discovery, and following). The other events (the presentation of the blank screen and color cards in irregular trials) were modeled as events of no interest. For correctly acquired rules, we modeled the onset of each colored card separately for all the three phases (i.e., rule search, rule discovery, and rule following) and convolved with the hemodynamic response function (HRF).

Next, we obtained three contrast images per participant, corresponding to the three conditions (i.e., the three phases) of interest and pooling across the four runs. These images were then subjected to a 1 × 3 full‐factorial ANOVA, for group‐level random effects statistical inference. The three phases, namely the rule‐search, rule‐discovery, and rule‐following phases, were compared against each other using the generalized linear model with separate subject predictors, and subjects were treated as random effects. Family‐wise error (FWE)‐corrected methods were used for multiple comparisons.

## 3 Results

**Figure 2:**
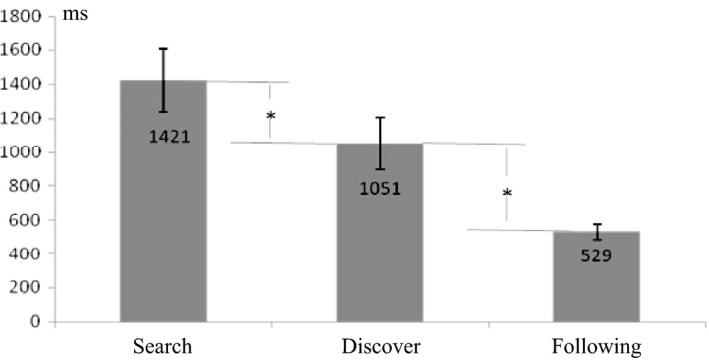
Different RTs at the three phases. Error bars indicate SD. *p < .001

Based on participants’ responses and the definition of rule discovery, we found that participants completed the task seriously and seemed to discover most of the predetermined rules. They discovered on average 19.1 rules (range: 17–20). The RT data for each of the three phases identified in correctly acquired rules are shown in Fig. 2. RT was significantly affected by phase type (*F* (1, 19)  = 150.3, *p *<* *.001), with the longest RT being observed for the rule‐search phase (1421 ms) and the shortest RT for the rule‐following phase (529 ms). Multiple comparisons indicated that the difference between the phases was statistically significant (*p *<* *.01).

**Figure 3:**
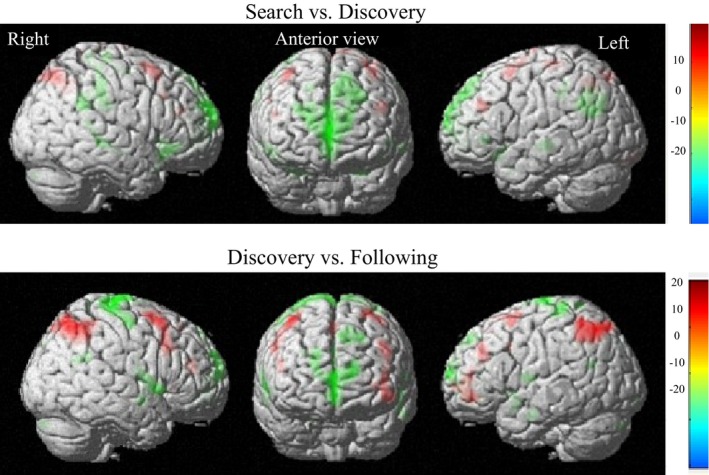
Whole‐brain statistical maps for regions exhibiting a significant difference between conditions. *Top*, the results of rule search minus rule discovery. *Bottom*, the results of rule discovery minus rule following. Red are the positive and the green are the negative results

The main aim of this study was to investigate whether different brain regions are active during rule search and rule discovery. Accordingly, we compared fMRI data obtained during rule search and rule discovery. As shown in Table 1 and Fig. 3, regions that were more active during rule search than during rule discovery lied in the posterior‐LPFC (BA 6, 9, 46: x = 54, y = 22, z = 38), the left precentral gyrus (x = −40, y = −26, z = 66), the bilateral precuneus (BA 7: x = 10, y = −74, z = 54), and the bilateral parietal lobule (BA 40: x = −36, y = −60, z = 48).

**Table 1:** Cerebral foci of activation for contrast of rule search and rule discovery

Anatomical localization	BA	MNI coordinates			Voxels	*T*_value_
		*x*	*y*	*z*		
More active for search than for discover						
L/R middle frontal gyrus	6/9/46	54	22	38	559	16.4
L precentral gyrus		−40	−26	66	65	9.87
L/R superior parietal lobule	40	−38	−58	50	349	9.33
L/R inferior parietal lobule	40	−36	−60	48	436	18.63
L/R precuneus	7	10	−74	54	470	17.18
L lingual gyrus	17/18	−8	−98	−14	108	11.78
More active for discover than for search						
R medial frontal gyrus	10	2	56	6	1542	21.12
R anterior cingulate	31/32	0	30	22	1085	16.39
L/R inferior frontal gyrus	47	50	30	−10	499	12.46
L/R superior frontal gyrus	8/10	18	48	46	1532	9.84
L/R superior temporal gyrus	21/22	−56	−62	24	412	10.26
L/R middle temporal gyrus	22	60	−30	−4	349	11.09
L/R para hippocampal gyrus	34	16	−4	−22	81	10.73
L amygdala	28	−18	−2	−26	42	10.73
L/R inferior parietal lobule	39/40	62	−34	40	390	12.05
R precentral gyrus	4	32	−26	74	109	9.29
R cerebellum posterior lobe		28	−86	−32	51	7.78

BA, Brodmann areas; L, Left; R, right.

Stereotactic MNI coordinates for significant clusters (FWE corrected, *p *<* *.001) given in millimeters with effect sizes (*t* scores) and cluster extent. In the voxels per cluster column, cluster extent is reported in correspondence of the main peak.

**Figure 4:**
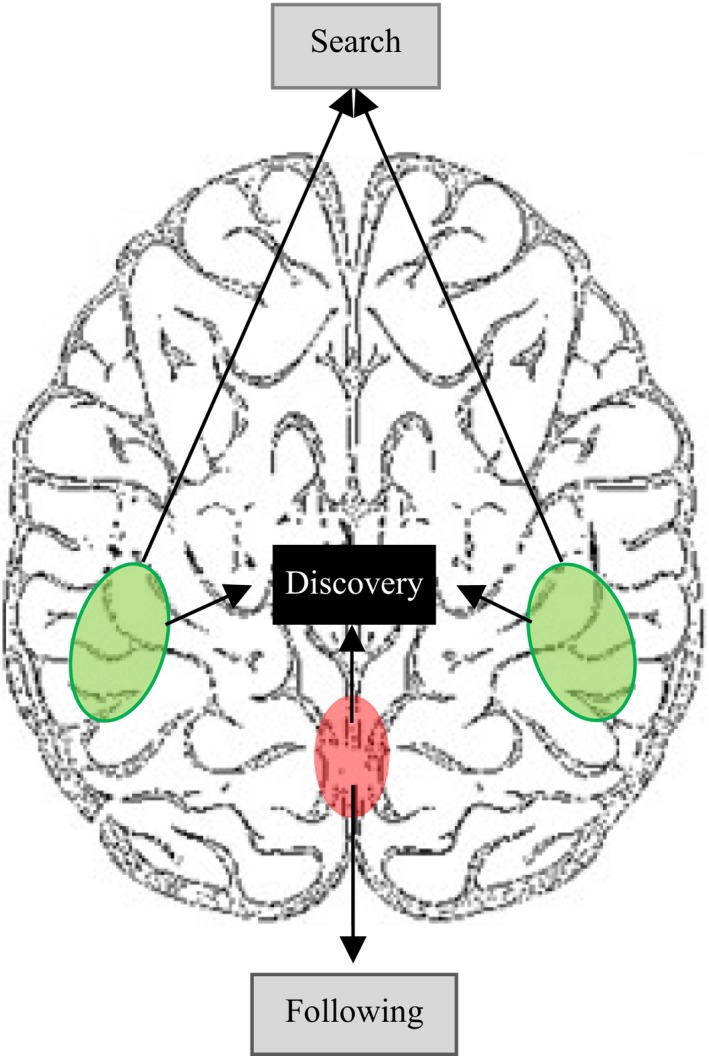
Illustration of the neural bases of the different phases of rule learning in frontal cortical areas. LPFC (green) contributes more to rule search, MPFC (red) contributes more to rule following, and these two regions both contribute to rule discovery that functioned as a “gate” to rule attainment

In contrast, a number of other areas were found to be more active during rule discovery than during rule search. As shown in Table 1 and Fig. 3, the largest region that was more active during rule discovery than rule search was the anterior MPFC, including the right medial frontal gyrus (BA 10: x = 2, y = 56, z = 6), right anterior cingulate (x = 0, y = 30, z = 22), and the bilateral superior frontal gyrus (BA 8, 10: x = 18, y = 48, z = 46). The bilateral inferior frontal gyrus (BA 47: x = 50, y = 30, z = −10), bilateral temporal gyrus, anterior part of the inferior parietal lobule (BA 39, 40: x = 62, y = −34, z = 40), as well as the bilateral parahippocampal gyrus, the left amygdala, and the right cerebellum were also more active during the rule‐discovery phase than the rule‐search phase.

In order to examine whether the brain basis of rule discovery was the same as the rule following, we also compared it with rule following. As shown in Table 2 and Fig. 3, the LPFC including the bilateral middle frontal gyrus (BA 6, 8, 10: x = −48, y = 46, z = −6), the left inferior frontal gyrus, and the left superior frontal gyrus, was more active during rule discovery than rule following. The bilateral parietal lobule was also more active during rule‐discovery phase as compared with rule‐following phase. In contrast, the MPFC including the bilateral superior frontal gyrus (BA 8, 10: x = 18, y = 60, z = 28), right anterior cingulate (BA 31, 32: x = 2, y = 30, z = 20) were more active during rule following than rule discovery. The temporal gyrus, parahippocampal gyrus, insula, and cerebellum were also more active in rule following than rule discovery.

**Table 2:** Cerebral foci of activation for contrast of rule discovery and rule following

Anatomical localization	BA	MNI coordinates			Voxels	*T*_value_
		*x*	*y*	*z*		
More active for discovery than for following						
L/R middle frontal gyrus	6/8/10	−48	46	−6	1152	19.83
L/R inferior frontal gyrus	47	38	24	0	136	7.59
L/R superior frontal gyrus	8	2	30	52	174	10.52
L/R inferior parietal lobule	40	−42	−60	52	709	19.73
L/R superior parietal lobule	7	36	−66	52	699	16.01
More active for following than for discovery						
R anterior cingulate	31/32	2	30	20	732	12.64
L/R superior frontal gyrus	8/10	18	60	28	961	8.82
L/R superior temporal gyrus	21/22	−58	6	−10	261	8.91
L/R middle temporal gyrus	21	60	−2	−10	116	8.69
L inferior temporal gyrus	21	−60	−6	−24	49	
R parahippocampal gyrus	34	16	−10	−26	88	8.15
R insula		48	6	−2	94	6.9
L/ R precentral gyrus	4/6	−60	−2	10	695	8.96
L/R cerebellum posterior lobe		−30	−86	−32	115	8.31
L/R precuneus	7	2	−58	38	866	14.09
L posterior cingulate	31	−4	−54	18	354	6.39

BA, Brodmann areas; L, Left; R, right.

Stereotactic MNI coordinates for significant clusters (FWE corrected, *p *<* *.001) given in millimeters with effect sizes (*t* scores) and cluster extent. In the voxels per cluster column, cluster extent is reported in correspondence of the main peak.

## 4 Discussion

Previous studies have attempted to identify the distinct brain areas that are activated during different phases of rule learning (Crescentini et al., 2011; Tachibana et al., 2009). However, the pattern of transient brain activities in response to rule discovery remains unclear. In this study, we adopted a modified rule learning task (Burgess & Shallice, 1996; Crescentini et al., 2011; Li et al., 2012), in which subjects were required to discover 20 different rules. We analyzed three distinct phases: rule search, rule discovery, and rule following (Fig. 4).

Consistent with recent findings by Li et al. (2012), we observed that the two subprocesses of rule acquisition, namely rule search and rule discovery, activate different brain regions. Compared with rule discovery, rule search evoked more activations in the posterior LPFC and parietal cortex. In contrast, the rule discovery evoked more brain activations than rule search in the anterior MPFC, parahippocampal gyrus, and temporal gyrus.

Previous studies revealed that the DLPFC was more active during rule acquisition (rule search and discovery) than during rule following (Seger & Cincotta, 2006). Consistent with previous studies, this study also found the significant contribution of DLPFC to rule acquisition as compared with rule following. The posterior DLPFC was found to be significantly activated in the rule‐search phase and the activation significantly decreased in the rule‐discovery phase and reached the lowest in the rule‐following phase. The decrease in activation in DLPFC from rule search to rule discovery and to rule following might reflect the hypothesis generation functions of DLPFC, that is, the process of searching for regularity among the stimuli or to generate possible hypotheses (Goel & Vartanian, 2005; Goel et al., 2007; Crescentini et al., 2011; Boettiger & D'Esposito, 2005; Reverberi et al., 2005). In this study, the hypothesis space decreased gradually from the rule‐search phase to the rule‐following phase. In the rule‐search phase, many possible hypotheses could be generated, and the hypothesis space was relatively large. However, in the rule‐discovery phase, the hypothesis space was restricted owing to the presence of a regular number/pattern of sequences. In the rule‐following phase, the generated hypothesis was further verified and uncertainty of the hypothesis was reduced (Crescentini et al., 2011). Therefore, the decreased activation of mid‐DLPFC from rule search to rule discovery may be associated with the decrease in uncertainty of the hypothesis (Huettel, Song, & McCarthy, 2005).

Compared with rule search, rule discovery significantly activated the anterior MPFC. The MPFC is located around the anterior cingulate sulcus (Rushworth, Walton, Kennerley, & Bannerman, 2004) and has been implicated in a diversity of functions, from reward processing and performance monitoring to the execution of control (Elliott, Dolan, & Frith, 2000; Niendam et al., 2012; Ridderinkhof, Ullsperger, Crone, & Nieuwenhuis, 2004; Shackman et al., 2011; Shenhav, Botvinick, & Cohen, 2013). MPFC is activated when an executed action is found to be inappropriate. A negative deflection (error‐related negativity, ERN) has been repeatedly observed in human electroencephalogram (EEG) studies (Gehring, Goss, Coles, Meyer, & Donchin, 1993; Miltner, Braun, & Coles, 1997), and similar MPFC activity has also been found in human functional magnetic resonance imaging (fMRI) studies, recording studies, and lesion studies (Behrens, Woolrich, Walton, & Rushworth, 2007; Gehring & Knight, 2000; Holroyd et al., 2004; Ito, Stuphorn, Brown, & Schall, 2003; Kolling, Behrens, Mars, & Rushworth, 2012; Mars et al., 2005; Procyk, Tanaka, & Joseph, 2000). In this study, the required behavioral action in the rule‐discovery phase was just to press a key indicating that the hidden rule had been correctly identified, and the wrong (e.g., false alarms) actions were not analyzed, so there is no inappropriate or error action in the moment of rule discovery. Therefore, MPFC activation observed in the rule‐discovery phase should not reflect the monitoring of error action of participants.

Location of the MPFC lies within the region that was also associated with social cognition (Overwalle, 2009). Nevertheless, we thought that the MPFC activity observed in our study is unlikely to be associated with the social cognition process, since there was no social interaction between participants and others (e.g., other people or animals). Before task, participants were instructed that they were tracing the movement of “a fictitious animal”. During task, the main cognitive process is encoding the numerical or spatial relationship between the current stimuli and the preceding stimuli, while the social interaction between participants and the imagined animal seemed to be unnecessary for the completion of the task.

On the contrary, MPFC activation in this study might reflect the action monitoring of external stimuli (i.e., the moving pattern of the blue circle). Specifically, during the rule‐discovery and rule‐following phases, participants should monitor the location of the blue circle and judge whether it was congruent with the generated hypothesis or the detected rule. As mentioned above, participants in the rule‐search phase might generate some hypotheses about the moving pattern of the blue circle (Crescentini et al., 2011). At the rule‐discovery phase, the critical function of the cognitive control is to monitor whether the moving action of the blue circle fits the hypothesis space. Similarly, during the rule‐following phase, the critical function of the cognitive control is to monitor whether the moving action of the blue circle follows the rule detected a few seconds ago. However, the rule might change unexpectedly, so the action monitoring is rather critical for the completion of the task with volatility. As compared with the rule‐search, in the rule‐discovery and rule‐following phases, there is no necessary to generate new hypothesis, because the generated hypothesis have been confirmed and the hidden rule had been revealed. Therefore, the main requirement in the rule‐discovery and rule‐following phases is to monitor the action of the moving blue circle. Correspondingly, the activation in LPFC decreased and the activation in MPFC increased during the rule trials, reflecting the function of MPFC in detecting and monitoring environmental volatility (Behrens et al., 2007; Hyman, Whitman, Emberly, Woodward, & Seamans, 2013).

Another function of MPFC in rule learning might be associated with the process of “grasping or comprehension” of the answer to a problem. Previous studies revealed increased levels of MPFC activation when subjects reflected on their own mental states (Amodio & Frith, 2006) or when task sets had to be internally generated as opposed to being fully externally cued (Forstmann, Brass, Koch, & von Cramon, 2005, 2006). Other studies on sentence completion or story comprehension also revealed that the anterior MPFC was activated when an appropriate completion of sentence was made, and found that more subjectively comprehensible stories elicited higher blood flow in the anterior MPFC (Maguire, Frith, & Morris, 1999; Nathaniel‐James & Frith, 2002). In this study, identification of the hidden rule during the rule‐discovery phase can also be classified as subjective comprehension, which is also likely to be intrinsically rewarding, and depends on making associations between information and flexible updating of these associations in light of incoming stimulus information (Elliott et al., 2000). Similar to sentence completion or story comprehension (Maguire et al., 1999; Nathaniel‐James & Frith, 2002), the experience of success in rule discovery might relate largely to a feeling of “grasping or comprehension”. During the rule‐following phase, the feeling of “grasping a rule” is further strengthened, which was reflected by the increased activation in the MPFC. When a rule was “grasped”, the rule information was stored in memory, and simultaneously activated the parahippocampal gyrus and temporal gyrus, which plays an important role in rule storage (Bunge, 2004; Crescentini et al., 2011; Donohue et al., 2005).

It is noticeable that there is huge body of literature using reversal learning tasks or Wisconsin Card Sorting Task (WCST) to explore the neural base of rule learning (Ghahremani, Monterosso, Jentsch, Bilder, & Poldrack, 2010; Hampshire 2006, 2012; Hornak et al., 2004; Monchi et al., 2001), but these studies seldom reported activity in the MPFC (BA10). Monchi et al. (2001) emphasized the feedback process and the card sorting after different types of feedbacks. Compared with positive feedback, negative feedback is a process of searching for new hypothesis and new dimension. They demonstrated that LPFC is more active for negative feedback as compared with positive feedback. In line with Monchi et al. (2001), the rule search in our study also activated the LPFC. It is necessary to note that the rule‐discovery process in Monchi et al. (2001) is corresponding to the first positive feedback. However, they did not compare the positive feedback‐related brain activation with that of the negative feedback. The possible reason is that they did not emphasize the discovery process. The reason why they did not pay attention to this process may be that the process of “rule discovery” is not easy to be distinguished from subjects’ speculation. Specifically, the essence of WCST is to find the correct classification rules. There are three perceptual dimensions in the experiment. Each perceptual dimension may be related to the current classification rule. So, the probability of guessing the correct answer at the first trial was 1/3. If the first guess is wrong, the probability of guessing the correct answer in the second trial is 50%. Obviously, in the WCST, the rule discovery is almost a process of trial and error, which is greatly dependent on speculation. In contrast, in our task, the criterion of “rule discovery” is based on three consecutive corrective responses. The probability of guessing the target rule is 1/2*1/2*1/2 = 1/8, that is, only a very small probability (12.5%) of discovering rule by guessing. Therefore, participants in our task had to carefully compare numerical or spatial relations between the consecutive numbers before they figure out the target rule.

Similarly, reversal learning tasks (e.g., Hampshire, Chaudhry, Owen, & Roberts, 2012) were often used to investigate the brain activation associated with the rule switching process. In the task, the subjects had to choose one of the three patterns that could produce reward (Hampshire et al., 2012). For the first choice, the percentage of accuracy is 33.3% based on random responses, and is 50% for the second choice. Subjects might approach to the rule at the third choice after the preceding two attempts. Accordingly, the third choice with a positive feedback can be defined as “rule discovery”. Actually, some participants were expected to find the target rule immediately after the presentation of the second negative feedback, using a method of exclusion. Perhaps, in the reversal learning task, it is not easy to clearly define the “rule‐discovery” process, so the researchers did not focus on this process. Taken together, previous WCST or reversal learning task did not specify the “rule‐discovery” process, so these studies did not report the brain activation in MPFC that was associated with the process of rule discovery.

Close to MPFC, the dorsal anterior cingulated (dACC) is also more active in rule discovery relative to in rule search. The dACC has been linked to outcome monitoring and behavioral adjustment (Kennerley, Walton, Behrens, Buckley, & Rushworth, 2006; Kolling et al., 2012; Amiez, Sallet, Procyk, & Petrides, 2012; Hayden, Pearson, & Platt, 2011; Rushworth & Behrens, 2008) in the studies on decision making. In our study, the stronger activity of dACC in rule‐discovery than that in rule‐search phase may reflect the function of cognitive control system involved immediately after finding out a rule. That is, the cognitive control system would require the subjects to adapt their behavior to the new situation (e.g., encountering the regular number sequences). In the rule‐search phase, the activation of LPFC is related to the process of searching for a hypothesis or rule. When the rule is searched, the cognitive control system is needed to monitor whether the current location of each new stimulus is congruent with the rule that has been discovered.

Finally, the result of this study confirmed that the brain activation associated with the transient process of rule discovery is different from that of rule search and rule following (Li et al., 2012). This raises the question of whether rule discovery should be classified under rule acquisition or rule implication phase, which is not easy to answer. Previous studies either regarded rule discovery as one part of rule acquisition (Crescentini et al., 2011; Tachibana et al., 2009), or did not classify it clearly (Badre et al., 2010; Reverberi et al., 2005; Seger & Cincotta, 2006; Strange et al., 2001). Based on the decision making and the external behavioral responses of subjects in this study, it would be appropriate to consider it as a part of the rule implication phase, because the judgment and the key pressing for rule‐discovery phase are the same as those for the rule‐following phase (i.e., both make a “it is congruent with a hidden rule” judgment by pressing the “yes” key), but differ from the key pressing action for the rule‐search phase (i.e., press the “no” key). Nevertheless, the cognitive process of rule discovery is not identical to that of rule following because neither the certainty of hypothesis nor the process of “breakthrough” in cognition is the same between these two phases. In brief, the hidden rule or regularity of stimuli was initially detected in the rule‐discovery phase, while the hypothesis regarding the identified rule was verified in the rule‐following phase. Based on the above discussion on the cognitive processing, behavioral response, and fMRI image analysis, we think it is proper to regard the rule‐discovery phase as a critical intermediate process in rule learning that serves as a “gate” between chaos and a regular path to follow. Another question is about the paradigm. This study adopt the rule attainment task that was used in previous studies, and found the different brain activation associated with different phases of rule learning. However, these effects were not dissociated from the passage of time. Further study is needed to address this issue.

It is necessary to note that the rule learning tasks used in this study essentially contain the components of inductive reasoning. Brain imaging studies on inductive reasoning have revealed somewhat different brain activations when different types of materials were used in the reasoning tasks. In the argument strength judgment task (Goel & Dolan, 2004; Goel, Gold, Kapur, & Houle, 1997; Liang, Goel, Jia, & Li, 2014), the left DLPFC was activated, reflecting the processing of semantic relationships between premises. However, the medial and lateral prefrontal cortices in the studies of Goel and colleagues were activated differently. The medial PFC was activated in Goel et al. (1997), whereas the lateral PFC was activated in Goel and Dolan (2004). The possible reason is that the arguments used in these two studies are different. In addition to the inferences from individual to individual, the arguments used in Goel et al. (1997) include inferences that require the integration of multiple relationships. For example, the following argument requires this integration: *Skeleton were dinosaurs, skeleton laid dense eggs; All dinosaurs laid dense eggs*. Goel et al. (1997) suggested that inductive generalization based on the integration of multiple relationships might be associated with activation in left medial PFC. The numerical inductive reasoning task used in Liang et al. (Jia, Liang, Shi, Wang, & Li, 2015; Liang, Jia, Taatgen, Borst, & Li, 2016; Liang, Jia, Taatgen, Zhong, & Li, 2014) is very similar to the rule learning task used in this study. These studies consistently found that the DLPFC was significantly activated during inductive reasoning (Jia et al., 2015; Liang, Jia, et al., 2014; Liang et al., 2016). However, since these studies did not distinguish between rule search and rule discovery, it is unclear which cognitive function is associated with the DLPFC activation. In combination with the results of this study, we speculate that the DLPFC activations observed in numerical inductive reasoning are more likely correlated with the process of rule search rather than rule discovery. The remaining concern is that these tasks also included rule discovery, but only one study reported medial PFC activation (Liang, Goel, et al., 2014), while others coherently reported lateral PFC activation, specifically the study of Jia et al. (2011) in which rule identification was explicitly required. The most likely reason might be that the medial PFC activation was relatively weaker than the strong activation in lateral PFC in the numerical inductive reasoning task.

In conclusion, this study provides evidence that the different subregions of PFC contribute differently to the different phase of rule learning. The lateral PFC was found to be more active during the rule‐search phase, in which relation information such as spatial and quantity relations are processed. During the rule‐discovery phase, the activation in LPFC decreased, while the activation in the MPFC increased significantly. The medial PFC were found to be more active during the rule‐following phase, reflecting the process of monitoring of the moving stimuli, and the mental state of “grasping a rule” or resolution of uncertainty.

## Funding Information

National Nature Science Funding, (Grant/Award Number: ‘31571118’).

## Conflict of Interest

None declared.
